# Preoperative cystourethroscopic evaluation of children with posterior hypospadias

**DOI:** 10.6026/973206300214995

**Published:** 2025-12-15

**Authors:** Zannatul Ferdous Peu, Samidur Rahman Md, Shaiful islam K.M, Umme Habiba Dilshad Munmun, Rabeya Khandaker, Kaniz Hasina

**Affiliations:** 1 Department of Paediatric Surgery, Bangladesh Medical University, Dhaka, Bangladesh; 2 Department of Paediatric Surgery, Dhaka Medical College Hospital, Dhaka, Bangladesh; 3 Department of Surgical Oncology, Ahsania Mission Cancer & General Hospital, Dhaka, Bangladesh; 4 Department of Pediatric Surgical Oncology, Dhaka Medical College Hospital, Dhaka, Bangladesh

**Keywords:** Proximal hypospadias, posterior hypospadias, cystourethroscopy, lower urinary tract anomalie

## Abstract

Posterior hypospadias is often associated with silent lower urinary tract anomalies that may affect surgical outcomes. Therefore, it is of interest to identify bladder and posterior urethral anomalies using cystourethroscopy. This observational study included 52 children with posterior hypospadias at Dhaka Medical College Hospital over 18 months. All patients underwent preoperative cystourethroscopic evaluation. Enlarged prostatic utricle was observed in 40.3% of patients, posterior urethral valve in 11.5%, and hydrodistention of ureteric orifices in 21.2%. Secondary bladder wall changes were noted in 32.7% of patients. Preoperative cystourethroscopy is essential for detecting lower urinary tract anomalies in posterior hypospadias. It helps guide surgical planning and may reduce postoperative complications.

## Background:

Hypospadias is one of the most common urogenital anomalies in male children, affecting about 1 in 200 live births. It results from incomplete development of urethral spongiosum and ventral prepuce, often with penile curvature of varying degrees [1]. Based on meatal location, the Duckett classification (1989) divides hypospadias into three groups: anterior (glanular, subcoronal, distal penile), middle (mid penile), and posterior (proximal penile, penoscrotal, scrotal, perineal) [2]. Severe hypospadias, especially posterior types, are more likely to have associated urinary tract anomalies. These include vesicoureteric reflux, bladder neck obstruction or hypertrophy, ureterocele, urethral duplication, enlarged prostatic utricle (EPU), and posterior urethral valve (PUV) [3]. Some minor anomalies, such as lower-grade prostatic utricles, cannot be detected on radiological investigations but can be identified by direct cystoscopy [4]. Enlarged prostatic utricle, PUV, or urethral duplication may persist silently and negatively affect posterior hypospadias repair [4, 5]. Posterior hypospadias repair is technically challenging, and additional anomalies may complicate outcomes [6, 7- 8]. Postoperative complications-including recurrent urinary tract infection, urinary obstruction, pseudo-incontinence, urethrocutaneous fistula, and failed urethroplasty-can be minimized by preoperative cystoscopic evaluation [5]. Cystoscopy before surgery allows direct visualization, reduces reliance on extensive radiology, and aids operative planning [9,10,11- 12]. Decisions regarding the surgical stage and technique depend largely on cystoscopic findings, as reported previously [4]. Therefore, it is of interest to show that cystourethroscopy can detect subtle bladder and urethral anomalies not seen on radiology, which can improve surgical planning and reduce postoperative complications in posterior hypospadias patients.

## Materials and Methods:

This was a cross-sectional type of observational study. The patients were chosen via purposive sampling. A total of 52 patients were included in this study. The study took place in the Department of Paediatric Surgery in Dhaka Medical College Hospital (DMCH), Dhaka, Bangladesh from September 2022 to February 2024.

## Inclusion criteria:

[1] All boys with bilaterally palpable testes having posterior hypospadias.

[2] Age between 1.5 years - 13 years.

## Exclusion criteria:

[1] Patients with diagnosed as Disorders of sex differentiation (DSD).

[2] Patients with other significant co morbidity such as- Chronic Kidney Disease (CKD), bleeding disorders.

[3] Patients whose parents were non-co-operative to allow to conduct the cystourethroscopic study.

The participants were selected on the basis of selection criteria from the patients of Posterior Hypospadias who were attended in the Department of Pediatric Surgery, DMCH in the above-mentioned time period. Purpose of the study was explained properly to every patient's guardian. Written informed consent was taken from every patient's guardian when they fulfilled the criteria. Relevant investigations (Complete Blood Count, Bleeding Time, Prothrombin Time, Activated Partial Thromboplastin Time, Serum creatinine, Ultrasonogram of Urinary System in symptomatic cases, Urine routine and microscopic examination, Urine Culture and Sensitivity) was evaluated in the above-mentioned places of study for pre anesthetic check-up. A data sheet was filled up during data collection. In each case information about the patient was collected in a prescribed questionnaire after getting informed written consent from the parents or legal guardians. Patient was prepared for procedure and kept nothing by Mouth for atleast 4 hours prior to procedure. A single shot of Injectable antibiotic Ceftriaxone as 50mg/kg/dose was given at the time of induction of anesthesia. After induction of anaethesia, painting and draping was done maintaining proper sterilization. All the parts of cystoscope were checked whether working properly or not. Lignocaine 2% jelly was used as lubricant to insert the cystoscope and Glycine 1.5% solution was used as bladder irrigation fluid. White balance was checked. Karl Storz size 8 or 9.5 fr caliber cystoscope was used according to the caliber of urethra with age maintaining proper sterilization. Findings were noted and collected in data sheet. Some videographic evidences were collected from cystoscope machine database. Any unusual event during procedure was noted duly. After cystourethroscopic evaluation, if no unfavorable anomalies were found, PH repair was done accordingly and post-operative care was taken. But if there was presence of any anomalies that needed correction, PH repair was postponed and anomalies were corrected in same setting or planned for next setting. All the data were compiled and sorted properly. The data analysis was conducted by Microsoft Excel version 16.88. The continuous variable was described using measures of central tendency, including mean, median and standard deviation whereas categorical variables were expressed as frequency and percentage. Ethical clearance for the study was taken from the Ethical Review Committee (ERC) of Dhaka Medical College prior to the commencement of this study. The aims and objectives of the study along with the risks and benefits of this study were explained to parents of each patient of study subjects in easily understandable local languages. An informed written consent was taken from every patient's legal guardian. Privacy, anonymity and confidentiality of the data information that identifying any patient was maintain strictly. Each participant enjoyed every right to participate or refuse or even withdraw from the study at any point of time.

## Results:

The Figure 5 (see PDF) represents age distribution of children with PH undergone preoperative cystoscopic screening where 61.5% of the patients were from 4.1 to 10 years, 28.9% in the range of 1.5 to 4 years and only 5 (9.6%) patients were in the range of 10.1 to 13 years. Table 1 (see PDF) shows that only 21 patients (40.3%) were found to have EPU of different grades (Figures 1 - see PDF & 2 - see PDF). Among them, 8 patients were found to have Grade I and same number was found to have Grade II EPU. Only 4 patients (7.7%) were found to have Grade III EPU. One patient was found to have Grade 0 EPU. More than half of the patient had no EPU. Table 2 (see PDF) shows only 6 (11.5%) out of 52 patients were diagnosed with Posterior Urethral Valve during cystoscopy and all of them had delayed presentation of the defect. Rest of the patients (88.5%) had no PUV (Figure 3 - see PDF). The data represented in the Table 3 (see PDF) shows that 59.6% patients had normal ureteric orifice as depicted as grade 0. Eleven patients (21.2%) patients were found to have H2 grade Hydrodistension of Ureteric Orifice (Figure 4 - see PDF). In addition to that, 9 patients had H1 grade ureteric orifice and only 1 patient was found to have H3 grade ureteric orifice. The Table 4 (see PDF) shows that only one patient (1.9%) out of 52 PH patients was found to have urethral duplication which was incomplete type of Urethral Duplication. Table 5 (see PDF) shows almost two third of the PH patients (67.3%) had no secondary bladder wall changes during preoperative cystoscopic screening. 16 (30.8%) patients showed trabeculation and only one (1.9%) out of 52 patients showed sacculation in bladder mucosa. Table 6 (see PDF) shows total 21 patients had EPU. One (9.1%) out of 11 proximal penile hypospadias patients had EPU. Similarly, 14(46.7%) out of 30, 3(42.9%) out of 7 and 3(75%) out of 4 patients respectively having penoscrotal, scrotal and perineal hypospadias had EPU. Two (18.2%) out of 11 Proximal hypospadias patients had PUV whereas 4(13.3%) out of 30 penoscrotal hypospadias patients had PUV. Four (36.3%) out of 11 proximal penile, 11(36.7%) out of 30 penoscrotal, 3(42.9%) out of 7 scrotal and 3(75%) out of 4 perineal hypospadias patients had higher grades of hydrodistention (H1, H2, H3). Only 1(3.3%) out of 30 penoscrotal hypospadias patient had urethral duplication. Three (27.3%) out of 11 proximal penile, 10(33.3%) out of 30 penoscrotal, 1(14.3%) out of 7scrotal and 3(75%) out of 4 perineal hypospadias patients had secondary bladder wall changes. The pattern of the cystoscopic findings shows the more severe the hypospadias is, the more incidences of associated anomalies are observed.

## Discussion:

This study of 52 posterior hypospadias patients aimed to detect lower urinary tract anomalies via cystoscopy. Associated anomalies are a known challenge for successful surgical repair. Undiagnosed pathology can negatively affect postoperative outcomes [5]. Common complications include poor stream, infection, and fistula formation [5]. Often, asymptomatic patients do not undergo routine uroradiological investigation [13]. However, recent articles show adverse outcomes in previously asymptomatic patients [5, 14]. Upon investigation, anomalies like enlarged prostatic utricle were found [5, 14]. Preoperative cystoscopy is a valuable diagnostic alternative in this context [5]. This evaluation revealed a variety of previously undiagnosed pathologies. An enlarged prostatic utricle was found in 21 patients. Of these, 12 cases were grade II or III, requiring surgical attention. Most of these patients also had associated hydrodistention of the ureteric orifices. Several also had secondary bladder wall changes. The presence of multiple findings suggests developed urinary stasis. A similar prevalence of surgically significant utricle has been reported previously [15]. Another recent study also reported a high rate of such anomalies [16]. These findings would likely harm repair outcomes if not corrected first. Undiagnosed lower urinary tract pathology is known to complicate repairs [5]. Six patients in this study had posterior urethral valves. All had bladder wall changes and higher-grade hydrodistention. A diagnostic dilemma existed due to their delayed presentation. A retrospective review found many patients were diagnosed with valves only after initial surgery [5]. Most had been asymptomatic beforehand [5]. This dual pathology complicates outcomes, requiring separate treatment [5]. Incomplete valves may remain silent until later. Twelve patients showed higher-grade hydrodistention of the ureteric orifice. This finding necessitated further evaluation for reflux. A prior study correlates this finding with higher-grade reflux [12]. Urinary stasis from primary anomalies likely causes this condition. One patient had an incidental finding of incomplete urethral duplication. Correction was required after diagnosis, a finding rarely reported [14]. Seventeen patients had secondary bladder wall changes. These were associated with other diagnosed anomalies like valves or enlarged prostatic utricle. Treating the primary cause positively affected these bladder changes. Twenty patients had no anomalies detected. The other 32 had one or more pathologies. This confirms that enlarged prostatic utricle and valves are primary drivers of other findings. Their prompt diagnosis and treatment may prevent future complications. Cystoscopy greatly assisted in planning subsequent treatment steps. No procedural complications occurred in this study, aligning with prior safety reports [4]. This study confirms that asymptomatic patients can harbor significant anomalies. These are readily diagnosed through preoperative cystoscopic screening.

## Conclusion:

We show that preoperative cystourethroscopic evaluation during posterior hypospadias surgery in children had been a handful of scope to diagnose associated urinary bladder and posterior urethral anomalies that could have perplexed the outcome of hypospadias surgery if not treated beforehand.
